# Familial Hypercholesterolemia Presenting With Joint Pain in an Adolescent Girl

**DOI:** 10.7759/cureus.106663

**Published:** 2026-04-08

**Authors:** Chaithra Potla, Dhivyalakshmi Jeevarathnam, Mahesh Janarthanan

**Affiliations:** 1 Department of Pediatrics, Sri Ramachandra Institute of Higher Education and Research, Chennai, IND; 2 Division of Pediatric Rheumatology, Department of Rheumatology, Sri Ramachandra Institute of Higher Education and Research, Chennai, IND

**Keywords:** childhood rheumatic diseases, familial hypercholesterolemia, family history, joint pain, xanthomas

## Abstract

Familial hypercholesterolemia (FH) is a genetic disorder causing high cholesterol levels from birth, significantly increasing the risk of early cardiovascular disease. This abstract summarizes the clinical presentation of FH in an adolescent girl who presented with musculoskeletal manifestations, progressive joint pain and cutaneous xanthomas, and retinal vessel changes. Investigations revealed significantly elevated low-density lipoprotein (LDL) cholesterol levels. Detailed family history revealed premature cardiovascular events and hypercholesterolemia in siblings and father. The patient was initiated on statin therapy, after which lipid levels improved and joint symptoms resolved.

## Introduction

Pediatric musculoskeletal complaints are sometimes the first clinical signal of underlying systemic pathology, often pre-dating more overt metabolic or cardiovascular markers. Joint pain in adolescents may be associated with inflammatory arthritides, chronic pain syndromes, benign hypermobility, and other causes [1,2]. Rarely, it can also manifest as a secondary complication of severe dyslipidaemia [3,4].

Familial hypercholesterolemia (FH), on the other hand, is an autosomal dominant condition characterized by elevated levels of total cholesterol (TC) and low-density lipoprotein (LDL) [5]. Patients with a homozygous state inheriting abnormal genes from both parents tend to have extremely elevated blood cholesterol levels as compared to heterozygous individuals who inherit only one defective gene. Most patients are diagnosed by the detection of elevated cholesterol levels or premature coronary artery disease [6]. Musculoskeletal manifestations have been described in patients with FH, including Achilles pain, tendinitis, oligoarticular arthritis, and polyarticular or rheumatic fever-like symptoms [7]. Tendinous xanthomas can mimic chronic synovitis, leading to progressive joint pain and restricted mobility. The pathogenesis of musculoskeletal manifestations in hyperlipidemia is not fully understood, but it may be an inflammatory synovitis or cholesterol crystal-induced arthropathy. Upto 38% children with FH can have joint symptoms, but musculoskeletal symptoms have been seldom described as the presenting manifestation in juvenile FH. Distinguishing these systemic mimickers from primary rheumatic diseases is vital as the treatment differs completely for the two conditions. This case emphasizes the need for meticulous systemic examination, a thorough family history, combined with a high index of suspicion for a timely diagnosis of life-threatening lipid disorders. In our case report, we have described the unique presentation of a child with FH presenting with arthralgia as the primary manifestation.

## Case presentation

**Figure 1:**
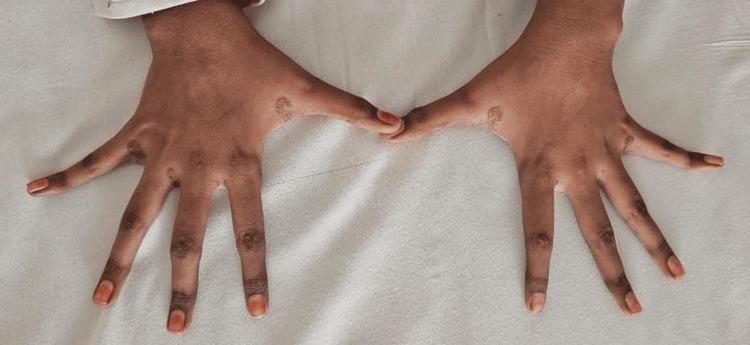
Xanthomas in interdigital spaces

**Figure 2:**
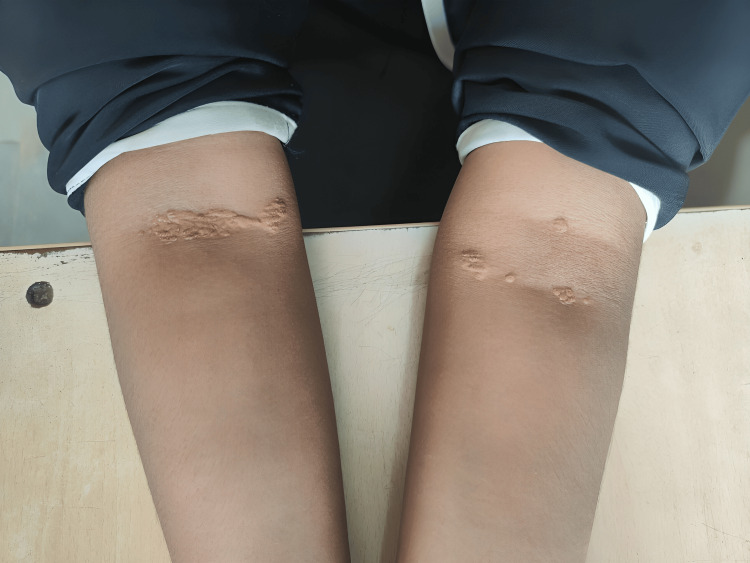
Xanthomas in the cubital fossa

**Figure 3:**
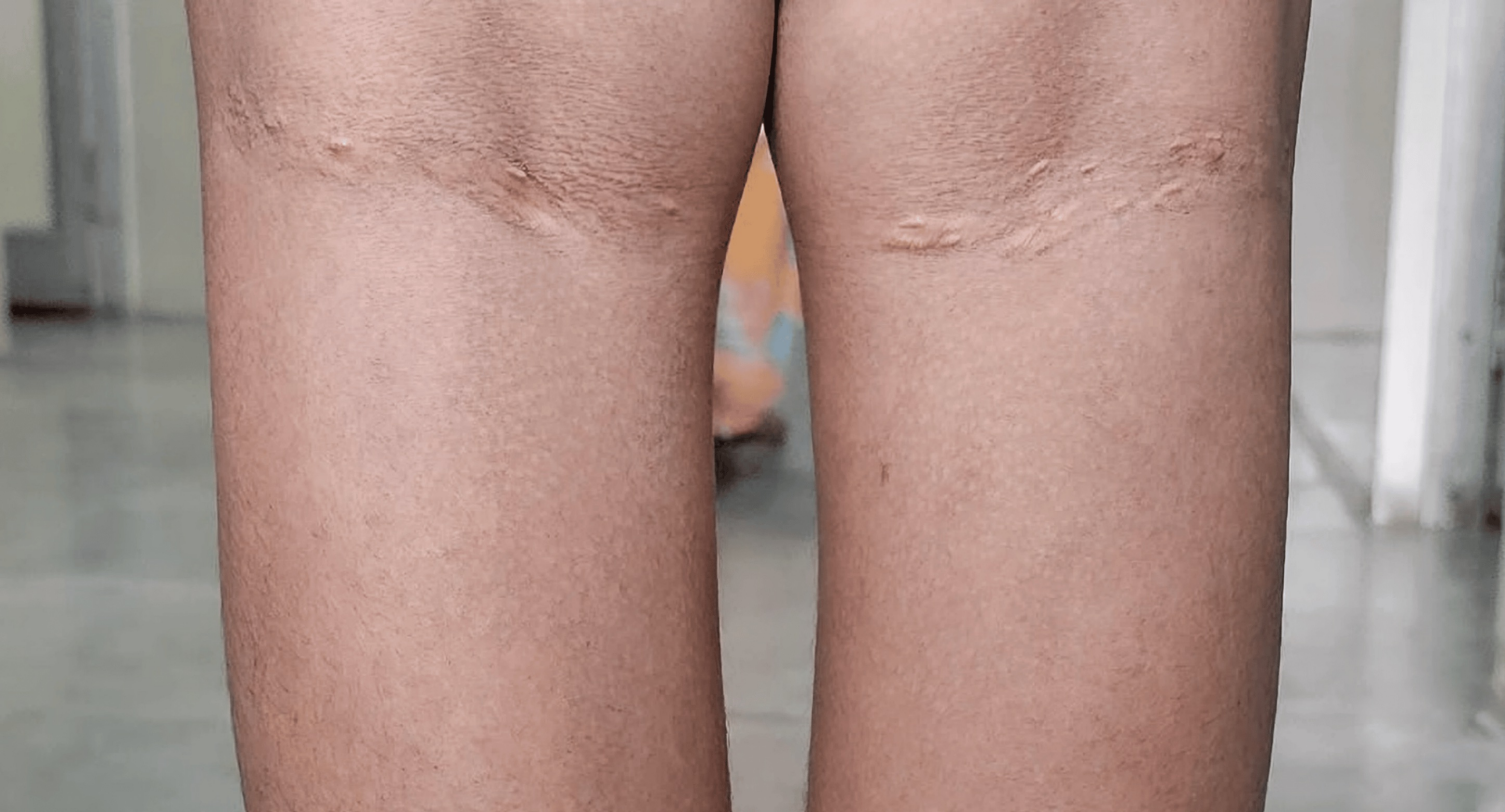
Xanthomas in the popliteal fossa

A 15-year-old female patient born of a third-degree consanguineous marriage presented with a seven-month history of pain over the bilateral knuckles, elbows, knees, and ankles. There was a progressive increase in pain symptoms, but no history of obvious swelling of joints, and the patient reported that there was no improvement with non-steroidal anti-inflammatory drugs. There were no other systemic symptoms like fever, rashes, oral ulcers, or alopecia. On examination, the child had yellow plaque-like lesions suggestive of xanthomas over the interdigital spaces of the hands (Figure 1), in the cubital fossae of both elbows (Figure 2), and over both popliteal fossae in the legs (Figure 3). Musculoskeletal system examination was normal except for bilateral Achilles tenderness. Blood pressure was normal. Examination of the eye revealed bilateral retinal vessel tortuosity. Blood investigations showed serum cholesterol levels of 598 mg/dl (High >240 mg/dl), serum triglycerides of 109 mg/dl (High 200-499 mg/dl), serum High-density lioprotein (HDL) of 21 mg/dl (High risk <45 mg/dl) and serum LDL cholesterol levels of 514 mg/dl (Very high >190 mg/dl). Cholesterol-HDL ratio was 28.5. Echocardiogram revealed mild tricuspid, mitral, and aortic regurgitation. Thyroid function test, serum insulin, glycated hemoglobin (HbA1C), fasting blood sugar, and postprandial blood sugar levels were within normal limits (Table 1). Rheumatoid factor and antinuclear antibody were negative. A detailed family history revealed significant history suggestive of metabolic diseases, with the mother being diagnosed with hypercholesterolemia previously, and the father had died of myocardial infarction at 35 years of age. There was also a history of the sudden death of two apparently healthy elder siblings at 19 and 17 years of age. A genetic test could not be performed, as the family could not afford it. Considering the skin manifestations, the family history, and laboratory investigations showing elevated serum cholesterol and LDL cholesterol, a diagnosis of FH was considered. The patient was started on atorvastatin 10 mg twice a day and was also advised dietary and lifestyle modifications. Her joint symptoms were resolved on follow-up at six months, and repeat blood tests at six months revealed total cholesterol of 382 mg/dl, triglycerides of 120 mg/dl, HDL of 26 mg/dl, and LDL cholesterol of 402 mg/dl.

**Table 1:** Summary of investigations

INVESTIGATION	RESULT	REFERENCE RANGE
Serum cholesterol	598mg/dl	Desirable <200mg/dl, Borderline high 200-239mg/dl, High >240mg/dl
Serum triglycerides	109mg/dl	Desirable <150mg/dl, Borderline 150-199mg/dl, High 200-499mg/dl
High-density lipoprotein (HDL) cholesterol	24mg/dl	No risk >65mg/dl, Moderate risk 45-65mg/dl, High risk <45mg/dl
Low-density lipoprotein (LDL) cholesterol	514mg/dl	Optimal <100mg/dl, Borderline high 130-159mg/dl, High 160-189mg/dl, Very high >190mg/dl
Cholesterol/HDL ratio	28.5	
Fasting glucose	88mg/dl	Normal: 70-110mg/dl
Fasting insulin	11.21microIU/ml	Normal: 2.6-24.9 microIU/ml
HbA1C (glycated hemoglobin)	5.7%	Normal <5.7%
FT3 (free triiodothyronine)	4.94pg/ml	Normal: 2-4.4 pg/ml
FT4 (free thyroxine)	2.61ng/dl	Normal: 0.93-1.7 ng/dl
TSH (thyroid-stimulating hormone)	0.049 microIU/ml	Normal: 0.4-6.4 microIU/ml

## Discussion

Inborn metabolic diseases cause the accumulation of toxic substances in the blood and organs due to an enzymatic deficiency or as a downstream of a production defect. FH is a lipid metabolism disorder characterized by a sustained elevation in blood levels of LDL cholesterol [8]. The most common form is autosomal dominant and is associated with mutations in the encoding genes for LDLR. Other mutations known to occur are APOB, PCSK9, and LDLRAP1, the latter causing severe disease with homozygous autosomal recessive inheritance [9]. Adults with familial FH tend to be diagnosed between the ages of 40 and 49 years, with more than one in six adults already having established atherosclerotic cardiovascular disease. Clinical manifestations developing earlier, along with arterial disease before the age of 10 years is characteristic of the homozygous form [10]. Approximately 40% patients affected by FH present with joint pain around 18-20 years of age. Recurrent episodes of subacute pain in Achilles tendons is one of the main manifestations, usually resolving spontaneously within 2-3 days [11]. The diagnosis of FH is based on clinical findings, family history, lipid levels, and genetic tests, if available. Our patient also satisfied Simon Broome diagnostic criteria with extremely elevated lipid levels and xanthomas and Dutch Lipid Clinic network diagnostic criteria for FH with very high elevated lipid levels (8 points), tendon xanthomas (6 points), and family history (1 point), securing much more than the 8 points required for diagnosis [12,13]. Considering the early onset of skin manifestations, extremely elevated levels of LDL cholesterol, and history of multiple deaths in the family at a young age, this child could have most likely had a homozygous form of the disease, but a genetic workup could not be done due to financial constraints. FH in children presenting with musculoskeletal manifestations and nodular swelling in joints and tendons, misdiagnosed as inflammatory arthritis, has been previously reported [4].

Mainstay treatment includes dietary and lifestyle modifications and statins, with the addition of ezetimibe to lower LDL cholesterol levels. The recommended target is < 135 mg/dl (< 3.5 mmol/L) for children above 10 years of age. PCSK9 inhibitors, Evolocumab and Alirocumab, could be used in patients who are not responding to the maximum tolerated dose of statin and for those at high risk of cardiovascular events [14-16]. Xanthomas usually do not respond to standard statin therapy and may require monoclonal antibody treatment for complete resolution [17]. Monoclonal antibody therapy has been found to be effective and safe in the treatment of FH in children; however, it is expensive, as long-term therapy is needed [18].

## Conclusions

Articular symptoms may be a rare presenting manifestation of FH in children. Our case report highlights the importance of family history and clinical examination, which might give a clue to the underlying systemic disease. The absence of typical signs of inflammation usually present in juvenile rheumatic diseases, negative serological tests, and non-response to non-steroidal anti-inflammatory medications should prompt the search for an alternative diagnosis. Genetic tests, though helpful in confirming the diagnosis, are not always feasible, either due to non-availability or due to financial constraints in resource-poor settings. In such situations, the diagnostic criteria discussed in our report may help in making the diagnosis. Early diagnosis and treatment are vital to prevent mortality from possible premature cardiovascular events.
